# Medications for Treating Alcoholism

**Published:** 1994

**Authors:** Raymond F. Anton

**Affiliations:** Raymond F. Anton, M.D., is professor of psychiatry and director of alcohol medication studies at the Medical University of South Carolina, Center for Drug and Alcohol Programs, Department of Psychiatry and Behavioral Sciences, Charleston, South Carolina

## Abstract

Researchers are evaluating medications to assist in treating alcoholism. Such medications may be used to treat withdrawal or co-occurring psychiatric disorders or as an adjunct to psychosocial treatment.

The prospect of using medications to help treat certain aspects of alcoholism^1^ appears increasingly attractive. Two developments have contributed to stimulating research in this area. First, there is an improved understanding of how brain cells use chemical messengers (neurotransmitters) to communicate, thereby producing mental states and, ultimately, behavior (see sidebar, p. 266). Second, it has been postulated that abnormalities in certain neurotransmitter systems may be common to both alcoholism and depressive disorders (see the article by Miller, pp. 261–264). This suggests that medications effective in treating psychiatric disorders may be useful for treating alcoholism as well. Researchers therefore have a theoretical basis for developing new medications as well as further evaluating a large number of existing medications for use in alcoholism treatment. Medications that prove useful in treating aspects of alcoholism can serve as adjuncts to standard psychosocial methods of treatment.

**How Nerve Cells Communicate**

The brain is a communication center. Nerve cells in the brain send messages to one another and to other cells in the body, thereby controlling all bodily processes, including thought, emotions, and behavior. Alcohol exerts its effects on mood and behavior by interfering with nerve cell communication. For the most part, the communicating ends of different nerve cells do not touch each other but are separated by a microscopic gap called a synapse. A nerve cell transmits a message by releasing a chemical called a neurotransmitter into the synapse, to be taken up by the receiving nerve cell. The message-receiving surface of a nerve cell is studded with protein molecules called receptors. There are several types of receptors, each of which recognizes and binds to a specific type of neurotransmitter. A given neurotransmitter molecule conforms to its receptor as a key fits into a lock.

The binding of a neurotransmitter to the appropriate receptor sets off a chain of chemical events within the receiving nerve cell. These events ultimately result in the promotion or inhibition of specific functions of that nerve cell.

Any step in the communication process is a potential site of action for psychiatric medications or for drugs, including alcohol, that affect the brain. Medications that mimic or enhance the effects of a neurotransmitter at a receptor are called agonists. Medications that block or inhibit the normal function of a receptor are called antagonists.

Neurotransmitters of particular importance to alcoholism treatment include dopamine, serotonin, and various opiates. Dopamine is involved in reward and pleasure mechanisms as well as movement coordination. Serotonin modulates the effects of other neurotransmitters; among its many functions are those relating to sleep, aggression, appetite, and mood, including mood disorders. Opiate neurotransmitters are similar to morphine, codeine, and other natural and synthetic opiates that relieve pain and produce a sensation of pleasure.

—Raymond F. Anton

After various preliminary studies, the ultimate test of an experimental medication is the controlled clinical trial, in which the effects of the medication may be compared with the effects of a medically inactive substance (placebo). An additional strategy is the double-blind trial, in which both subjects and therapists remain ignorant of the treatment administered so that results can be interpreted with minimal bias.

In addition to clinical effectiveness, new medications for treating alcoholism should ideally meet special safety and compliance criteria. Such medications should not

- interact harmfully with alcohol
- have a significant abuse potential
- be toxic to organs such as the liver, which already may have been damaged by long-term alcohol consumption
- have unpleasant side effects that might limit their acceptability with patients whose motivation already may be minimal.

This article briefly discusses medications intended to reduce craving, treat psychiatric disorders, discourage consumption, antagonize alcohol’s effects, and ameliorate withdrawal symptoms. (For more detail, see Meyer 1989; Kranzler and Orrok 1989; Litten and Allen 1991; Naranjo and Sellers 1992; Kranzler and Anton in press).

## Anticraving Medications

Scientists believe that the positively reinforcing effects of alcohol (see sidebar, p. 267) are related to the release of dopamine into a brain area called the nucleus accumbens (Koob 1992). Dopamine release is controlled by several other neurotransmitters, including serotonin and the naturally occurring opiates. Although the specific roles of these neurotransmitters are unclear, medications that modify their actions may alter the craving for alcohol (table 1).

**Reinforcement and Craving**

Research has suggested mechanisms by which alcohol affects brain cells to bring about a state of alcohol dependence (alcoholism) in susceptible people. For example, the actions of alcohol on certain brain centers may lead to sensations perceived as rewarding, or pleasurable. The process by which a person learns to repeat rewarding behavior is called positive reinforcement. This process encourages the development of alcoholism in persons who are vulnerable for underlying genetic or psychosocial reasons (Wise and Rompre 1989).

In addition, alcohol tends to relieve stress and anxiety. The process by which a person learns to avoid behavior that causes unpleasant sensations is called negative reinforcement. Some people may have a genetically based or learned stress intolerance (Sher and Levenson 1982) that alcohol ameliorates, leading to negative reinforcement.

The positive and negative reinforcing effects of alcohol are associated with subjective feelings of craving coupled with a loss of control over alcohol consumption. Craving is not easily defined or quantified because it is a state of mind. Loss of control consists of initiating drinking despite obvious negative consequences or drinking more than is intended. By modifying the links between reward, stress, and craving, medications potentially could reduce the urge to drink and the loss of control over drinking. As such, this would “tip the balance” so that psychosocial treatments could be more effective.

—Raymond F. Anton

ReferencesSherKJLevensonRWRisk for alcoholism and individual differences in the stress-response-dampening effect of alcoholJournal of Abnormal Psychology913503671982714257310.1037//0021-843x.91.5.350WiseRARomprePPBrain dopamine and rewardAnnual Review of Psychology40191225198910.1146/annurev.ps.40.020189.0012032648975

### Opiate Antagonists

The most promising medication for treating alcoholism to date is naltrexone (ReVia™), an opiate antagonist. In two studies, naltrexone reduced the relapse rate to heavy alcohol consumption by about 50 percent in alcoholics over a 12-week period (Volpicelli et al. 1992; O’Malley et al. 1992); all subjects received additional psychosocial treatment (see the article by Volpicelli et al., pp. 272–278).

Other opiate antagonists also are being examined for effectiveness. For example, a small placebo-controlled study found that nalmefene decreased the rate of relapse to abusive drinking in alcoholics (Mason et al. 1994).

### Medications That Affect Serotonin

In addition to its role in brain reward pathways such as the nucleus accumbens (McBride et al. 1991), serotonin may affect the urge to drink indirectly through its role in impulse control and mood stabilization. Serotonin also may alleviate the stress intolerance caused by mood disturbances, thereby reducing the influence of environmental experiences on drinking behavior.

A series of studies conducted primarily at the Addiction Research Foundation in Toronto, Canada, examined serotonin-enhancing medications in a group of heavy alcohol consumers with minimal alcohol-related problems (Naranjo and Bremner 1992). Medications, such as citalopram, zimelidine, and fluoxetine (Prozac^®^), that increase the amount of serotonin at the synapse (see sidebar, p. 266) produced a 10-to 20-percent decrease in alcohol consumption, measured mainly as drinks consumed per day. However, it is unclear whether these medications would be as effective in more severely affected alcoholics.

Although citalopram and zimelidine are not available in the United States, fluoxetine is used widely here as an anti-depressant. This medication is relatively safe, has fewer serious side effects than many other antidepressants, and lacks abuse potential (Mendels 1987). In a study of a group of 20 alcoholic men given free access to alcohol in a locked hospital ward for 28 days, fluoxetine lowered alcohol consumption 14 percent during the first week but not subsequently (Gorelick and Paredes 1992). The influence of psychosocial factors on these results is unknown. The researchers concluded that response to medication might vary in different settings. Kranzler and colleagues (in press) found that fluoxetine combined with coping skills therapy did not reduce alcohol relapse and other drinking behavior in outpatient alcoholics.

Certain selective serotonin receptor antagonists have decreased alcohol consumption in preliminary studies (Meert 1993). These medications include ondansetron (Zofran^®^), used to control nausea and vomiting in patients undergoing cancer chemotherapy, and ritanserin, which has been used to treat anxiety. Ondansetron has been reported to reduce daily alcohol consumption by about 20 percent, compared with placebo, in actively drinking, nonseverely alcohol-dependent patients (Sellers et al. 1994). A large multisite study is examining ritanserin in combination with cognitive behavioral therapy in outpatient alcoholics.

### Medications That Affect Dopamine

Interestingly, few studies have examined the effect on alcoholism of medications that affect the dopamine system directly. One study utilizing the dopamine agonist bromocriptine showed a reduction of craving and alcohol use (Borg 1983). The dopamine antagonist tiapride has shown promise for promoting abstinence in recovering severe alcoholics during the immediate postdetoxification period (Shaw et al. 1994).

Progress in this area has been limited by the significant number of adverse side effects associated with existing medications that affect dopamine. Additionally, some medications, such as amphetamine, that stimulate the release of dopamine into the synapse are potentially addictive.

### Other Medications

Preliminary results from European studies indicate that calcium homotaurinate reduces alcohol consumption and craving (Lesch et al. 1994; Poldrugo et al. 1994). The mechanism of this action is unknown but may involve an effect on the brain chemicals glutamate and taurine.

## Medications to Treat Alcoholics With Psychiatric Disorders

Comorbidity is the occurrence of two or more illnesses in the same person. Recent large-scale population studies have confirmed significant comorbidity of psychiatric disorders and alcohol use disorders (Kessler et al. 1994; Regier et al. 1990; for more information, see the article by Miller, pp. 261–264). Researchers are attempting to develop medications to treat psychiatric comorbidity (Anthenelli and Schuckit 1993) on the theory that certain psychiatric conditions may lead to loss of control over alcohol consumption. Although this assumption is controversial, medications under study are generally safe and effective for the psychiatric conditions, and many show promise in animal studies for reducing alcohol consumption (table 2).

### Anxiety Disorders

Many alcoholics display symptoms of excessive or inappropriate anxiety. A commonly prescribed antianxiety medication is buspirone (Buspar^®^), which appears to exert its effect by raising serotonin concentrations in the brain. Because an increase in serotonin may lead to a decrease in alcohol consumption (Gorelick and Paredes 1992; Meert 1993), buspirone might be useful for treating alcoholics with comorbid-generalized anxiety disorders. In addition, buspirone has no abuse or addiction liability, in contrast to the benzodiazepine tranquilizers (Valium^®^ and others), which are not recommended for long-term treatment of alcoholics (Malcolm et al. 1992).

Controlled trials of buspirone in anxious alcoholics have shown inconsistent results. Buspirone decreased both anxiety and alcohol consumption in alcoholic outpatients who also were treated with coping skills therapy (Kranzler et al. 1994). However, buspirone alone may not be effective for alcoholic inpatients, whose anxiety and alcoholism both tend to be more severe than that of alcoholic outpatients (Malcolm et al. 1992).

Panic disorder, a chronic, often debilitating anxiety disorder, is found in a significant subgroup of alcoholics who may drink to alleviate the symptoms (Lepola 1994; George et al. 1990). Imipramine (Tofranil^®^), an antidepressant sometimes used to treat panic disorder, is under investigation for treating panic disorder in alcoholics.

### Depression

Depression may be more common than anxiety in alcoholics. The majority of depressive symptoms in alcoholic patients are alcohol induced and abate shortly after abstinence (Brown and Schuckit 1988). However, a substantial minority of alcoholics suffer from intermittent major depressive episodes, chronic low-grade depressions, or manic-depressive disorder (see the article by Miller, pp. 261–264). Few studies have examined the treatment of these comorbid conditions.

In a preliminary 12-week study using imipramine, 45 percent of a group of alcohol abusers and alcoholics with depressive disorders showed improvement in both mood and drinking behavior. However, these subjects represented only 32 percent of the patients who started the trial, since a number of patients dropped out prior to receiving an adequate dose of medication (Nunes et al. 1993). In a double-blind trial, the antidepressant desipramine (Norpramin^®^) improved depressive symptoms and reduced drinking days in depressed alcoholics when compared with a placebo (Mason and Kocsis 1991).

Lithium, prescribed mainly for manic-depression, often is used to treat alcoholics with mood disorders (Lejoyeux and Ades 1993; Fawcett et al. 1987). During the mid-1980’s, the Veterans’ Administration sponsored a large multisite trial of lithium in depressed and non-depressed alcoholics (Dorus et al. 1989). This 12-month study of 171 alcoholics with current or past histories of major depression or chronic low-grade depression found no significant effect of lithium on depressive symptoms, alcohol consumption, or rate of alcohol relapse. As in many of the studies conducted to date, subjects were not required to attend psychosocial therapy, although almost one-half did so for some time during the trial. In addition, manic-depressives were excluded from participating in the trial, and not all depressed patients were depressed when the study was initiated. Nevertheless, the study suggests that lithium is not useful as a general treatment for alcoholism in either depressed or nondepressed alcoholics.

Fluoxetine was discussed previously as a potential anticraving medication. Preliminary studies suggest that it might reduce drinking and depressive symptoms in alcoholics with major depression (Cornelius et al. 1993).

### Posttraumatic Stress Disorder

Posttraumatic stress disorder (PTSD) is a poorly understood and complex disorder that often occurs in alcoholics. It has been suggested that the symptoms of PTSD can be accounted for by a deficiency of opiate neurotransmitters (van der Kolk et al. 1989), possibly leading to an increased sensitivity of opiate receptors. Alcohol consumption has been shown to elevate opiate neurotransmitter levels. This suggests the possibility that persons with PTSD may be more sensitive to alcohol’s rewarding or stress-reducing effects. Researchers therefore are investigating the use of naltrexone to normalize opiate receptor sensitivity in these patients.

In addition, because PTSD often is associated with depression, researchers are examining the effect on alcohol consumption of antidepressants that increase serotonin in the synapse. In combination with these studies, researchers are providing patients with psychosocial therapy aimed at PTSD.

## Aversive Medications

Medications that cause an unpleasant (aversive) reaction when taken with alcohol have long been a mainstay of alcoholism treatment. These medications generally interfere with the metabolism of alcohol by the liver, permitting a noxious chemical (acetaldehyde) to accumulate in the blood. Consuming alcohol after taking an aversive medication results in such symptoms as rapid heartbeat, shortness of breath, headache, nausea, and vomiting, thereby discouraging further alcohol consumption (table 3).

### Disulfiram

The most widely used and studied aversive medication is disulfiram (Antabuse^®^). Despite favorable results from preliminary studies, a large, multisite, double-blind controlled clinical trial conducted by the Veterans’ Administration (Fuller et al. 1986) found disulfiram ineffective for maintaining sobriety over an extended period. These negative findings may reflect the failure of many patients to take the medication as prescribed. Those patients who consumed alcohol but remained in the study and continued to take their disulfiram significantly decreased their alcohol consumption.

Disulfiram may be effective in highly motivated persons who take the medication over an extended period of time, especially in situations in which compliance can be monitored (Chick et al. 1992). In addition, clinicians prescribe disulfiram for short-term use in patients who have developed some internal control over their craving but who desire additional, external protection to face a temporary high-risk situation.

Some researchers have attempted to develop a long-acting disulfiram formulation that could be injected or incorporated into a timed-release surgical implant, thereby eliminating the problem of daily medication compliance (Philips 1992). These attempts have not yet been successful. In addition, the safety and patient acceptability of long-acting disulfiram formulations have not been clarified (Allen and Litten 1992).

### Calcium Carbimide

Calcium carbimide produces an aversive reaction to alcohol similar to that produced by disulfiram. In a study by Peachey and colleagues (1989), the 69 alcoholics completing the 4-month controlled study were abstinent on at least 85 percent of the days, and 58 percent remained abstinent throughout the study. However, the reduction of alcohol consumption with calcium carbimide was not significantly greater than with placebo. As noted in previous studies, motivation and compliance are thus key factors in evaluating medications taken under controlled conditions in which support for abstinence is high.

### Daidzin

National headlines recently focused on the discovery of a new compound, extracted from the ubiquitous southern vine kudzu, that was found to inhibit alcohol consumption by hamsters (Keung and Vallee 1993). This investigation was prompted by the knowledge that kudzu was used in ancient China (600 A.D.) to treat alcohol abuse. However, the compound’s efficacy in treating alcoholism remains to be proven.

## Alcohol Antagonists

The concept of medications that would act as “sobering up pills” has captured the interest of clinicians and the public alike. In the emergency room, where intoxicated patients pose a variety of diagnostic and behavioral problems, a medication capable of reversing intoxication would be of immense clinical value. An alcohol antagonist also might be used to treat patients who have ingested potentially lethal amounts of alcohol. Unfortunately, experimental medications capable of antagonizing alcohol tend to cause anxiety or promote seizures. Moreover, doses capable of causing apparent sobriety may not protect the patient against alcohol’s behavioral or medical effects. The patient might still be at risk of driving while impaired or may suffer dangerous adverse health effects through increased drinking in an attempt to overcome the antagonist’s effects (Lister and Nutt 1988). Therefore, issues of toxicity and legal liability may limit the development of such medications.

Unlike most other chemicals that affect the brain, alcohol does not have a specific receptor site to which it attaches. Therefore, chemists have been unable to develop an antagonist to directly inhibit or reverse alcohol’s actions. However, some medications indirectly antagonize alcohol’s effects on certain brain cell functions. The opiate antagonist naloxone, for example, has been reported to reverse the life-threatening coma caused by very large amounts of alcohol (Sorensen and Mattisson 1978); this effect has not been replicated.

Theoretically, medications that block alcohol’s short-term effects might be effective over longer periods as well. For example, lithium, at levels used to treat mood disorders, blocks the feeling of intoxication from alcohol doses that produce blood levels at about the level defining legal intoxication in most States (Judd et al. 1977; Judd and Leighton 1984). A similar finding has been reported for thyrotropin-releasing hormone, a natural substance released from the pituitary gland that can be taken intravenously as a medication. The mechanism of this effect is unknown but may involve a general increase in arousal, counteracting alcohol’s sedative effect (Knutsen et al. 1989).

## Treatment of Alcohol Withdrawal

Alcohol withdrawal syndrome occurs in heavy drinkers shortly after cessation of a drinking bout. Symptoms range from profuse sweating, rapid heart beat, elevated blood pressure, tremors, agitation, and anxiety to seizures, disorientation, and hallucinations (Gorelick and Wilkins 1986). Medications to treat withdrawal generally modify brain processes, often at receptors. (For more detail, see Castaneda and Cushman 1989; Schultz 1991; Anton and Becker in press.)

### Benzodiazepine Sedatives

The most frequently used medications for treating moderate to severe alcohol withdrawal are sedatives of the benzodiazepine class, especially diazepam (Valium) and chlordiazepoxide (Librium^®^). In recent years other medications in this class, such as oxazepam (Serax^®^) and lorazepam (Ativan^®^), also have become widely used for this purpose. The latter medications are particularly useful in patients with alcoholic-induced liver damage, who have a diminished ability to metabolize diazepam and chlordiazepoxide.

Benzodiazepines may cause excessive sedation and have some addiction liability. Medications that share some of the effects of benzodiazepines on nerve cells, but which may have less abuse potential because of their slightly different chemical structure, are undergoing investigation.

### Medications That Affect Norepinephrine

Alcohol withdrawal symptoms appear to result, at least in part, from overactivity of nerve cells that communicate using the neurotransmitter norepinephrine. Therefore, not surprisingly, medications that decrease norepinephrine activity have been found useful in treating alcohol withdrawal. Among these medications are clonidine (Catapres^®^) and propranolol (Inderal^®^), both commonly prescribed to treat high blood pressure. These medications do not cause as much mental confusion or sedation as do benzodiazepines. They also do not induce pleasurable effects that might lead to addiction. In contrast to benzodiazepines, however, clonidine and propranolol do not block the development of seizures, the most serious potential withdrawal symptom (Anton and Becker in press).

### Antiseizure Medications

The severity of withdrawal symptoms tends to increase progressively in alcoholics who have undergone repeated episodes of withdrawal (Ballenger and Post 1978; Brown et al. 1988; Booth and Blow 1993). This phenomenon may be analogous to a form of brain sensitization called kindling—small electrical currents, individually too small to produce a seizure, will ultimately produce a seizure if applied repeatedly over time. Therefore, medications that inhibit kindling have been examined for treating alcohol withdrawal.

One such medication is carbamazepine (Tegretol^®^), commonly prescribed for epileptic seizures. Carbamazepine has been found in well-controlled studies to be as effective as benzodiazepines for treating alcohol withdrawal (Malcolm et al. 1989). It is reasonably safe and lacks abuse liability. More research is needed to determine whether the use of carbamazepine during the first episodes of withdrawal will diminish the risks of more serious symptoms during subsequent withdrawals. Carbamazepine probably is most suited for alcoholics who have experienced a previous seizure, irrespective of its relationship to alcohol withdrawal, and for patients who already have had multiple medication-assisted detoxifications from alcohol (Brown et al. 1988).

## Future Directions

Several issues must be addressed in future research on the use of medications to treat alcoholism. Among these is the optimal strategy for combining the use of medications with psychosocial treatment. Annis (1991) postulated that alcoholism treatment targeted toward relapse prevention should proceed in two stages. In the first stage, medication—for example, to reduce craving, increase stress tolerance, or ameliorate a mood disturbance—is combined with a cognitive social learning therapy intended to strengthen the patient’s internal controls.

If patients attribute their success solely to their medication, they may feel excessively vulnerable to relapse when the medication is discontinued. Therefore, in the second stage, after craving and mood fluctuations are reduced, cognitive social learning therapy allows the patient to attribute abstinence to self-initiated behavioral change, increased coping skills, and enhanced motivation.

Because the concomitant use of medications and psychosocial treatment is likely to be more costly than each applied alone, it will be necessary to establish the cost-effectiveness for these approaches in the new era of efficient health care delivery. Questions regarding the type of training needed to implement these strategies, as well as the length of treatment, will need to be examined during the cost-benefit analysis.

## Figures and Tables

**Table 1:** Studies of Medications for Treating Alcoholism

Class	Patient Type^1^	Efficacy
**Serotonergic**		
Citalopram*	Heavy drinker	Weakly efficacious
Zimelidine*	Heavy drinker	Weakly efficacious
Fluoxetine	Heavy drinker	Weakly efficacious
	Alcohol dependent	Mixed results (i.e., some positive and some no efficacy)
Sertraline	Alcohol dependent	Ongoing
Ritanserin*	Alcohol dependent^2^	Weakly efficacious
	Alcohol dependent	Ongoing
Ondansetron	Alcohol dependent	Weakly efficacious

**Opiate Antagonists**		
Naltrexone	Alcohol dependent	Strongly efficacious^3^
Nalmefene*	Alcohol dependent	Ongoing

**Others**		
Lithium	Alcohol dependent	Weak to no efficacy
Bromocriptine	Alcohol dependent	Weakly efficacious
Tiapride*	Alcohol dependent	Moderately efficacious
Calcium homotaurinate*	Alcohol dependent	Moderately/strongly efficacious

NOTE: In a double-blind trial, both subjects and therapists remain ignorant of the treatment administered so that results can be interpreted with minimal bias. All studies are double blind except as noted.

1 The terms included under patient type (i.e., “heavy drinker” and “alcohol dependent”) are defined differently in different studies.

2 Preliminary, not double-blind, study.

3 Volpicelli et al. 1992; O’Malley et al. 1992.

* Medication is not available commercially.

SOURCES: Selected references are provided. Full citations of these studies are available from the author.

**Table 2:** Studies of Medications for Treating Alcoholics With Psychiatric Disorders

Class	Psychiatric Disorders	Efficacy
**Serotonergic**		
Buspirone	General anxiety	Mixed results (i.e., some positive and some no efficacy)
Fluoxetine	Depression	Ongoing
Sertraline	Depression	Ongoing
	Posttraumatic stress disorder*	Ongoing

**Noradrenergic**^1^		
Desipramine	Depression	Weakly efficacious
Imipramine	Depression*	Weakly efficacious
	Panic disorder	Ongoing

**Mood Stabilizers**		
Lithium	Depression	Weak to no efficacy
Valproate	Manic-depressive disorders*	Weakly efficacious

**Others**		
Naltrexone	Posttraumatic stress disorder	Ongoing

NOTE: In a double-blind trial, both subjects and therapists remain ignorant of the treatment administered so that results can be interpreted with minimal bias. All studies are double blind except as noted.

1 Noradrenergic medications affect the neurotransmitter norepinephrine.

* Preliminary, not double-blind, study.

SOURCES: Full citations of these studies are available from the author.

**Table 3:** Studies of Alcohol-Sensitizing Medications

Class	Patient Type	Study Design	Efficacy
Disulfiram (oral)	Dependent	Preliminary	Positive efficacy
	Dependent	Double blind^2^	Mixed results (i.e., some positive and some no efficacy)
	Dependent	Preliminary/supervised^3^	Positive efficacy
	Dependent	Controlled/supervised^3^	Strongly efficacious^4^
Disulfiram (implanted)^1^	Dependent	Controlled	Weak to no efficacy
Calcium carbimide^1^	Dependent	Double blind	Weak to no efficacy

NOTE: In a double-blind trial, both subjects and therapists remain ignorant of the treatment administered so that results can be interpreted with minimal bias.

1 Medication is not available commercially.

2 Multisite study.

3 Subjects were monitored to ensure medication compliance.

4 Chick et al. 1992.

SOURCES: Full citations of these studies are available from the author.

## Footnotes

1 Throughout this article, terms such as “alcohol abuse,” “alcoholism,” and “heavy drinking” are used. There is overlap among these terms in the alcoholism literature; therefore, the wording in each case is based on the terms used in the reference cited.
